# Obesity and Mortality Among Patients Diagnosed With COVID-19: A Systematic Review and Meta-Analysis

**DOI:** 10.3389/fmed.2021.620044

**Published:** 2021-02-05

**Authors:** Tahmina Nasrin Poly, Md. Mohaimenul Islam, Hsuan Chia Yang, Ming Chin Lin, Wen-Shan Jian, Min-Huei Hsu, Yu-Chuan Jack Li

**Affiliations:** ^1^Graduate Institute of Biomedical Informatics, College of Medical Science and Technology, Taipei Medical University, Taipei, Taiwan; ^2^International Center for Health Information Technology (ICHIT), Taipei Medical University, Taipei, Taiwan; ^3^Research Center of Big Data and Meta-Analysis, Wan Fang Hospital, Taipei Medical University, Taipei, Taiwan; ^4^Department of Neurosurgery, Shuang Ho Hospital, Taipei Medical University, Taipei, Taiwan; ^5^Professional Master Program in Artificial Intelligence in Medicine, Taipei Medical University, Taipei, Taiwan; ^6^Research Center for Artificial Intelligence in Medicine, Taipei Medical University, Taipei, Taiwan; ^7^Taipei Neuroscience Institution, Taipei Medical University, Taipei, Taiwan; ^8^School of Health Care Administration, Taipei Medical University, Taipei, Taiwan; ^9^Graduate Institute of Data Science, Taipei Medical University, Taipei, Taiwan; ^10^Department of Dermatology, Wan Fang Hospital, Taipei, Taiwan; ^11^TMU Research Center of Cancer Translational Medicine, Taipei Medical University, Taipei, Taiwan

**Keywords:** COVID-19, SARS-CoV-2, obesity, body mass index (BMI), mortality

## Abstract

Coronavirus disease 2019 (COVID-19) has already raised serious concern globally as the number of confirmed or suspected cases have increased rapidly. Epidemiological studies reported that obesity is associated with a higher rate of mortality in patients with COVID-19. Yet, to our knowledge, there is no comprehensive systematic review and meta-analysis to assess the effects of obesity and mortality among patients with COVID-19. We, therefore, aimed to evaluate the effect of obesity, associated comorbidities, and other factors on the risk of death due to COVID-19. We did a systematic search on PubMed, EMBASE, Google Scholar, Web of Science, and Scopus between January 1, 2020, and August 30, 2020. We followed Cochrane Guidelines to find relevant articles, and two reviewers extracted data from retrieved articles. Disagreement during those stages was resolved by discussion with the main investigator. The random-effects model was used to calculate effect sizes. We included 17 articles with a total of 543,399 patients. Obesity was significantly associated with an increased risk of mortality among patients with COVID-19 (RR_adjust_: 1.42 (95%CI: 1.24–1.63, *p* < 0.001). The pooled risk ratio for class I, class II, and class III obesity were 1.27 (95%CI: 1.05–1.54, *p* = 0.01), 1.56 (95%CI: 1.11–2.19, *p* < 0.01), and 1.92 (95%CI: 1.50–2.47, *p* < 0.001), respectively). In subgroup analysis, the pooled risk ratio for the patients with stroke, CPOD, CKD, and diabetes were 1.80 (95%CI: 0.89–3.64, *p* = 0.10), 1.57 (95%CI: 1.57–1.91, *p* < 0.001), 1.34 (95%CI: 1.18–1.52, *p* < 0.001), and 1.19 (1.07–1.32, *p* = 0.001), respectively. However, patients with obesity who were more than 65 years had a higher risk of mortality (RR: 2.54; 95%CI: 1.62–3.67, *p* < 0.001). Our study showed that obesity was associated with an increased risk of death from COVID-19, particularly in patients aged more than 65 years. Physicians should aware of these risk factors when dealing with patients with COVID-19 and take early treatment intervention to reduce the mortality of COVID-19 patients.

## Introduction

### Rationale

The outbreak of coronavirus disease 2019 (COVID-19), caused by severe acute respiratory coronavirus 2 (SARS-CoV-2), has spread globally and created mounting concern (1). Healthcare organizations and providers are trying to find solutions to reduce the spread of disease and fatality rates. The rapid increase in the number of cases has created an unbearable burden on the healthcare system, especially in developing counties where healthcare systems are more fragile (2, 3). Early diagnosis of severe patients is essential to improve patient conditions and reduce mortality. Earlier classification of mild and severe COVID-19 patients could facilitate the proper utilization of limited resources (4). Multiple studies reported changes in several laboratory parameters [(e.g., the number of lymphocytes, C-reactive protein (CRP), interleukin-6 (IL-6), and erythrocyte sedimentation rates (ESR)] in the COVID-19 patients, but data are not sufficient to show their correlation according to severity and mortality (5, 6). Therefore, finding an appropriate risk factor is essential to classify mild and severe patients at an early stage.

Obesity is defined as abnormal fat accumulation and is a common, costly condition (7). According to the WHO report, obesity is classified into three groups based on body mass index (BMI). The prevalence of obesity has tripled since 1975 and is an established risk factor of other diseases such as diabetes, hypertension, heart disease, and cancers (8–10). Paradoxically, obesity has been shown to decrease mortality among patients diagnosed with pneumonia and ARDS (11–14). Recent epidemiological studies have shown obesity to be associated with both neutral and increased risk of mortality among patients diagnosed with COVID-19 (15, 16), but mechanisms underlying this are still unclear. Previous studies noted that the number of hospitalization and mechanical ventilation cases is higher in patients with obesity that can be associated with an increased rate of mortality (17, 18). Moreover, obesity is related to the downregulation of the inflammatory pathway, which leads to increase expression of inflammatory molecules, including interleukin-6 (IL-6). Age and elevated IL-6 were proclaimed as significant predictors of in-hospital mortality (19, 20).

### Goal of Investigation

The objectives of the current comprehensive and rigorous meta-analysis were to investigate relevant epidemiological studies for evaluating the association between obesity and mortality of COVID-19 patients. The findings of this study could help healthcare providers to take preventive actions and use early treatment strategies for these high-risk groups.

#### Research Aims

- To determine whether obesity is associated with an increased rate of mortality among the patients diagnosed with COVID-19.

- To calculate the strength of association between class I, class II, and class III obesity and mortality of COVID-19 patients.

- To elucidate the association between associated factors (e.g., diabetes, CKD, COPD, smoking) and mortality of COVID-19 patients.

## Methods

### Meta-Analysis Guidelines

The Preferred Reporting Items for Systematic Reviews and Meta-Analyses (PRISMA) was used to select potential study inclusion. Moreover, the Meta-analysis of Observational Studies in Epidemiology (MOOSE) guidelines were also considered for this study (21) (Supplementary Table 1).

### Search Strategy

We did a compressive systematic search to collect all relevant articles that evaluated the effect of COVID-19 on patients in terms of obesity and mortality. Any article published in English was considered for inclusion; an article search strategy was developed to retrieve all articles between January 1 and August 30, 2020. Articles search were conducted in the most popular electronic databases such as Scopus, PubMed, EMBASE, and Web of Science. The following search terms were used to retrieve articles: “Obesity” OR “BMI,” OR “Overweight” AND “mortality related to COVID-19” OR “death related to COVID-19” (Supplementary Table 2). We removed all duplicate articles, and a final search for relevant articles was performed on the reference list of retrieved articles.

### Selection Criteria

We developed a priori inclusion criteria and included articles of COVID-19 patients' mortality due to obesity, associated risk factors such as demographic factors, and comorbidities. Articles were included if they (a) were peer-reviewed, (b) were published in English, (c) were cohort or comparison design, (d) included patients with more than 20, (e) and reported effect size as odds ratio (OR), risk ratio (RR) or hazard ratio (HR). We excluded studies if they were published as a review or case series.

### Study Selection

Two authors (MdI and TP) screened titles and abstracts from the search results. Predefined selection criteria were used to select relevant full-text articles during the screening process. Afterward, all full-text articles were evaluated carefully for inclusion and data extraction. The same two authors (MdI and TP), however, independently evaluated each potential article for inclusion. Any disagreement during those screening process was resolved by the main investigator (Y-CL).

### Data Extraction

All the selected studies were then finally reviewed to extract potential information regarding obesity and mortality of patients with COVID-19 and associated risk factors. One author collected information about author name, publication years, study design, number of patients with COVID-19, number of alive patients and number of dead patients, percentage of mortality, risk factors, inclusion and exclusion criteria, and effect sizes.

### Quality Assessment

The Quality In Prognosis Studies (QUIPS) tool was utilized to examine the risk of bias (RoB), which is recommended by the Cochrane Prognosis Methods group (22). The QUIPS tool has six evaluation domains that are used to evaluate validity and bias in studies of prognostic factors: (a) study participation, (b) study attrition, (c) prognostic factor measurement, (d) outcome measurement, (e) study confounding, and (f) statistical analysis and reporting (23). RoB is classified into three groups: low, moderate, and high risk. The Grading of Recommendations Assessment, Development and Evaluation (GRADE) approach was considered to assess the confidence in the estimate of effect. GRADE was utilized to determine the quality of evidence based on several factors such as risk of bias, inconsistency, imprecision, indirectness, and publication bias.

### Statistical Analysis

The primary outcome was the mortality of patients with COVID-19 due to obesity, and secondary outcomes were increased risk of mortality among class I, class II, and class III obesity. In subgroup analyses, we also evaluated the risk of mortality associated with comorbidities, age, gender, and other factors. Risk ratios with 95%CIs were calculated from the HRs or ORs. We used a random-effects model to calculate the heterogeneity between studies. However, studies heterogeneity was assessed using Cochran Q statistics and inconsistency statistics (*I*^2^). We followed previous studies that considered heterogeneity as very low, low, medium, and high if I^2^ value 0~25%, 25–50%, 50~75%, and >75% (24, 25). The Forest plot was drawn to present effect size, and the funnel plot was drawn to present publication bias.

## Results

### Study Selection

**Figure 1:**
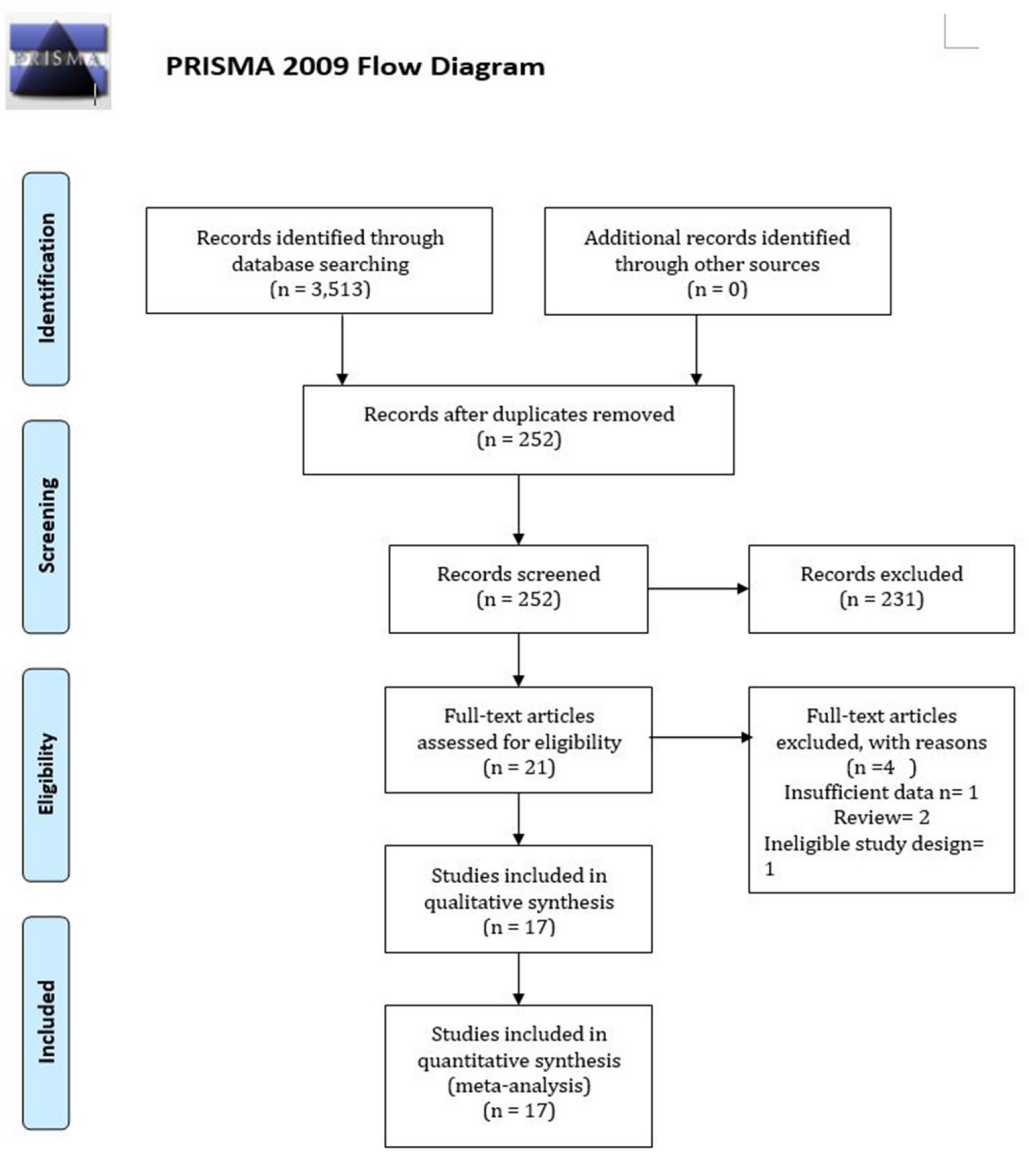
Flow diagram of the study selection process.

A total of 3,513 unique articles were retrieved after searching for electronic databases. Of those, 3,492 articles were excluded after reviewing their titles and abstracts because they have not fulfilled pre-specified selection criteria. Overall, 21 articles went to full-text review and were scrutinized for final inclusion. However, four studies were further excluded because they have been published in the form of a review. Finally, 17 articles met the inclusion criteria for meta-analysis (15, 16, 26–40). Figure 1 shows the overall study selection process.

### Study Characteristics

Table 1 shows the clinical characteristics of the included studies. Of those, 16 studies had a retrospective cohort study design, and one study had a prospective design. Eleven studies used data from North American patients, four studies used data from European countries (France, Italy, and the UK), and one study used data from 47 countries. The range of male patients was 43.3–80.2%. All of the studies used demographics, clinical variables.

**Table 1:** Characteristics of the included studies assessing the risk of mortality of COVID-19 patients with obesity.

**Author**	**Country**	**Design**	**Total** **sample**	**Male** **(%)**	**Age (mean/median)**	**Variable**	**Findings**
Bello-Chavolla et al. (33)	Mexico	Retrospective	177,133	57.70	63.5	Demographics, clinical, smoking	1.25 (1.17–1.34)
Klang et al. (35)	USA	Retrospective	3,406	56	76	Demographics, clinical, smoking	1.31 (0.91–1.89)
Nakeshbandi et al. (36)	USA	Retrospective	504	52	68	Demographics, clinical, smoking	1.30 (0.99–1.93)
Pettit et al. (37)	USA	Retrospective	238	47.5	58.5	Demographic, clinical, smoking	1.70 (1.10–2.61)
Palaiodimos et al. (29)	USA	Retrospective	200	49	64	Demographics, clinical, smoking, symptoms	3.78 (1.45–9.84)
Czernichow et al. (34)	France	Prospective	5,795	65.41	59.6	Demographics, and clinical	2.30 (1.77–2.98)
Hamer et al. (15)	UK	Retrospective	334,329	45.5	56.4	Demographics, biomarker, smoking	1.52 (0.94–2.44)
Rottoli et al. (38)	Italy	Retrospective	482	N/A	N/A	Demographic, clinical	12.10 (3.24–45.07)
Zhang et al. (40)	China	Retrospective	3,201	N/A	N/A	laboratory	1.35 (1.07–1.70)
Tartof et al. (31)	USA	Retrospective	6,916	44.98	49.1	Demographics, smoking, clinical characteristics	1.95 (1.09–3.49)
Price-Haywood et al. (16)	USA	Retrospective	3,481	43.3	55.5	Demographics, clinical, locations	0.99 (0.77–1.27)
Goyal et al. (39)	USA	Retrospective	1,687	60	66.5	Demographics, clinical, laboratory, smoking, in-hospital events	1.41 (0.73–2.69)
Halasz et al. (27)	Italy	Retrospective	242	80.2	64	Demographics, clinical, laboratory	1.99 (0.90–4.38)
Wang et al. (30)	USA	Retrospective	58	52	67	Demographics, laboratory, clinical	2.04 (0.49–8.36)
Hajifathalian et al. (28)	USA	Retrospective	770	61	63.5	Demographics, clinical, laboratory data, clinical outcomes	1.15 (0.61–2.13)
Kim et al. (26)	Multiple countries	Retrospective	2,491	53.2	62	Ethnicity, time of hospitalization, smoking status, clinical characteristics	1.09 (0.91–1.29)
Anderson et al. (32)	USA	Retrospective	2,466	58	67	Demographics, clinical	1.26 (1.03–1.54)

### Quality of Evidence

**Figure 2:**
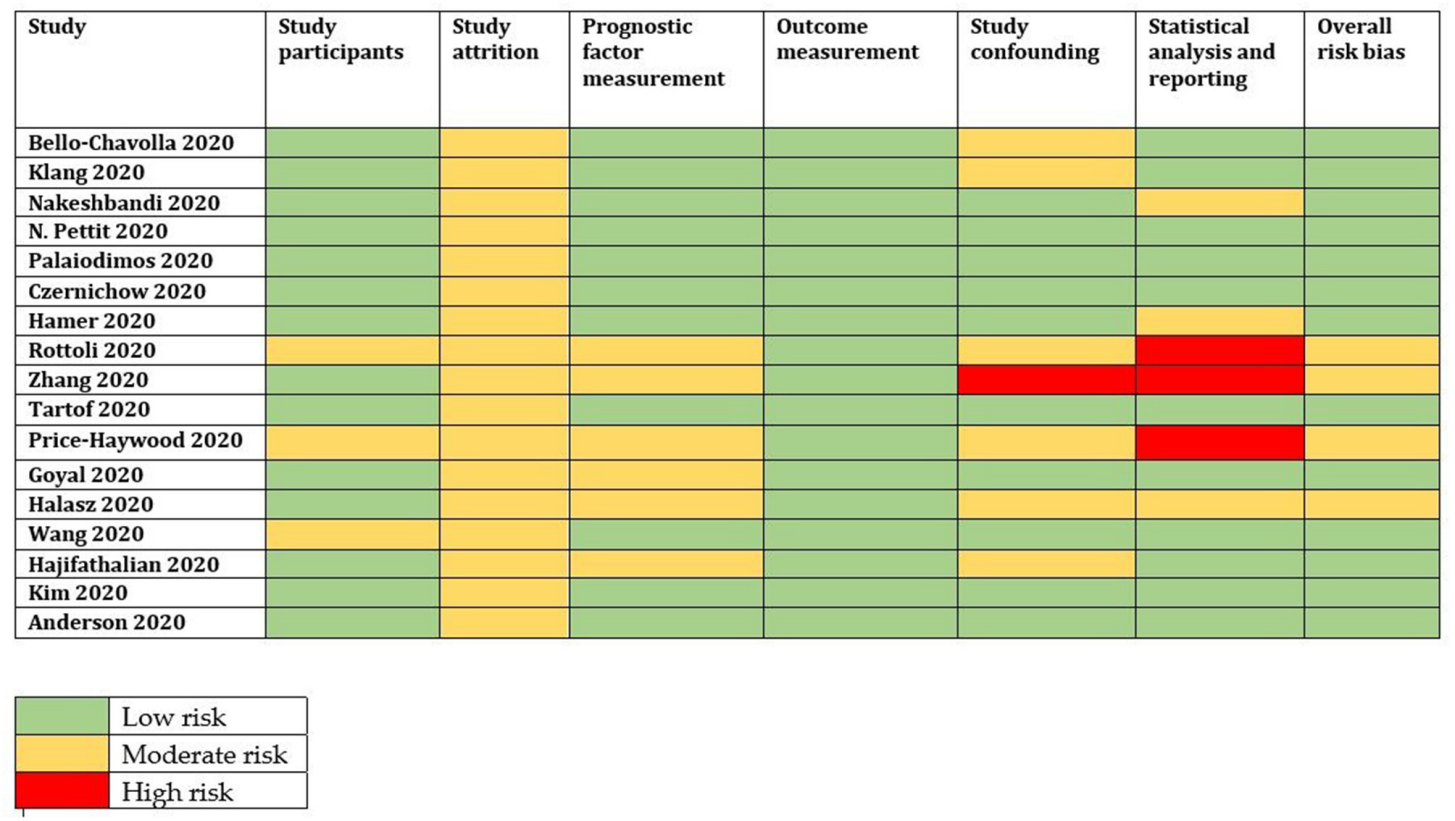
Risk of bias according to the QUIPS tool.

The QUIPS tool was used to evaluate the risk bias among the included studies. Thirteen studies were of low risk bias, and four studies were of moderate risk bias (Figure 2). The overall quality of evidence is strong in the meta-analysis. Supplementary Table 3 presents a summary of the GRADE evidence profile for our meta-analysis. For the increased rate of mortality among obese patients, the certainty was “low.”

### Primary Outcome

#### Obesity and Mortality of Patients With COVID-19

**Figure 3:**
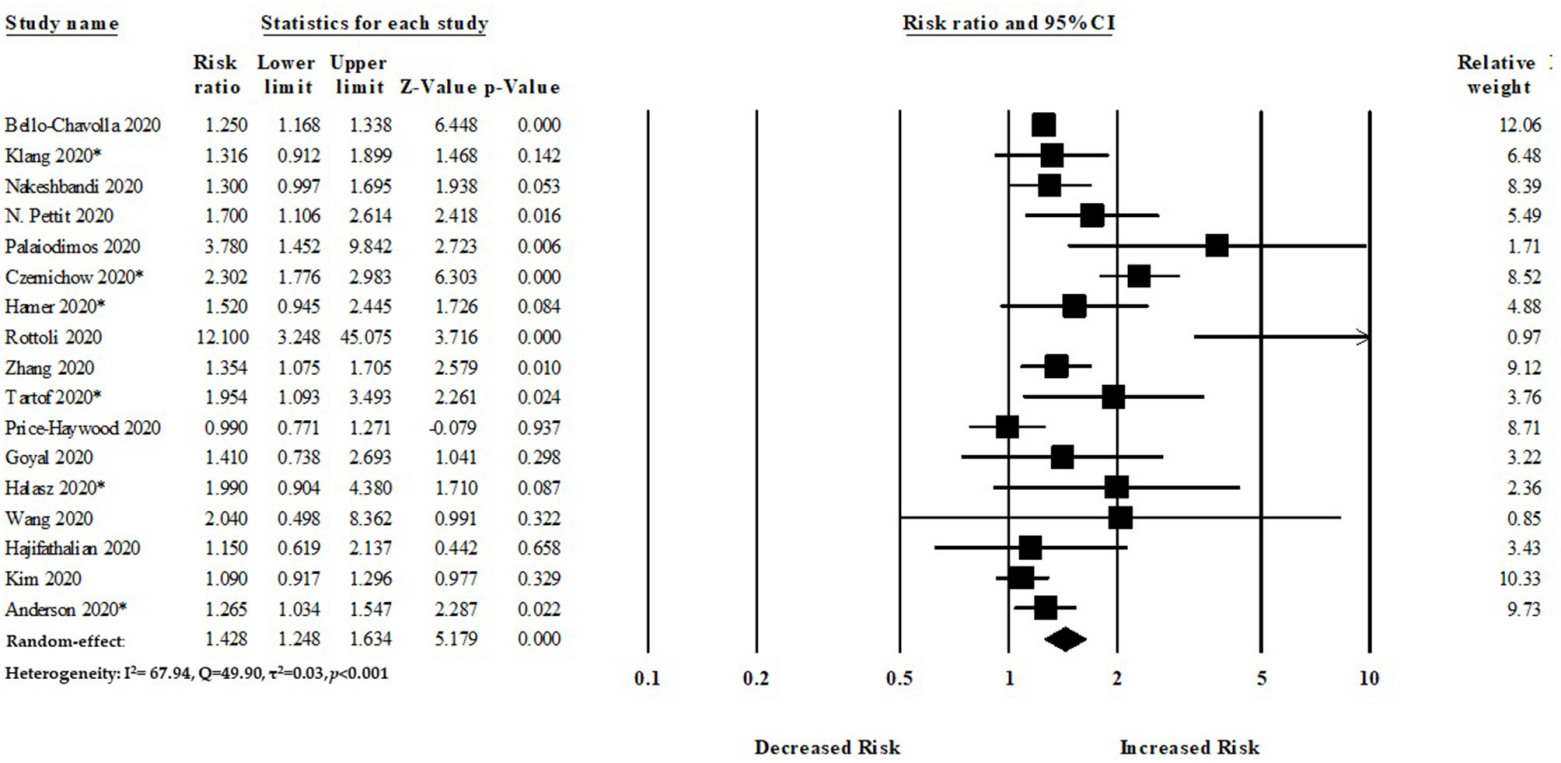
Association between obesity and risk of mortality among COVID-19 patients.

Of the 17 studies considered in our meta-analysis, 16 studies reported a significantly increased risk of mortality among patients with COVID-19, and one study reported a decreased risk of mortality. All the studies adjusted their effect size with potential variables to reduce potential bias. Collectively, our meta-analysis suggested a significantly increased risk of mortality with obesity, with a pooled RR of 1.42 (95%CI: 1.24–1.63, *p* < 0.001) (Figure 3). The *I*^2^ statistics among the studies was 67.94%, indicating a moderate risk of heterogeneity.

### Secondary Outcome

#### Types of Obesity and COVID-19 Mortality

**Figure 4:**
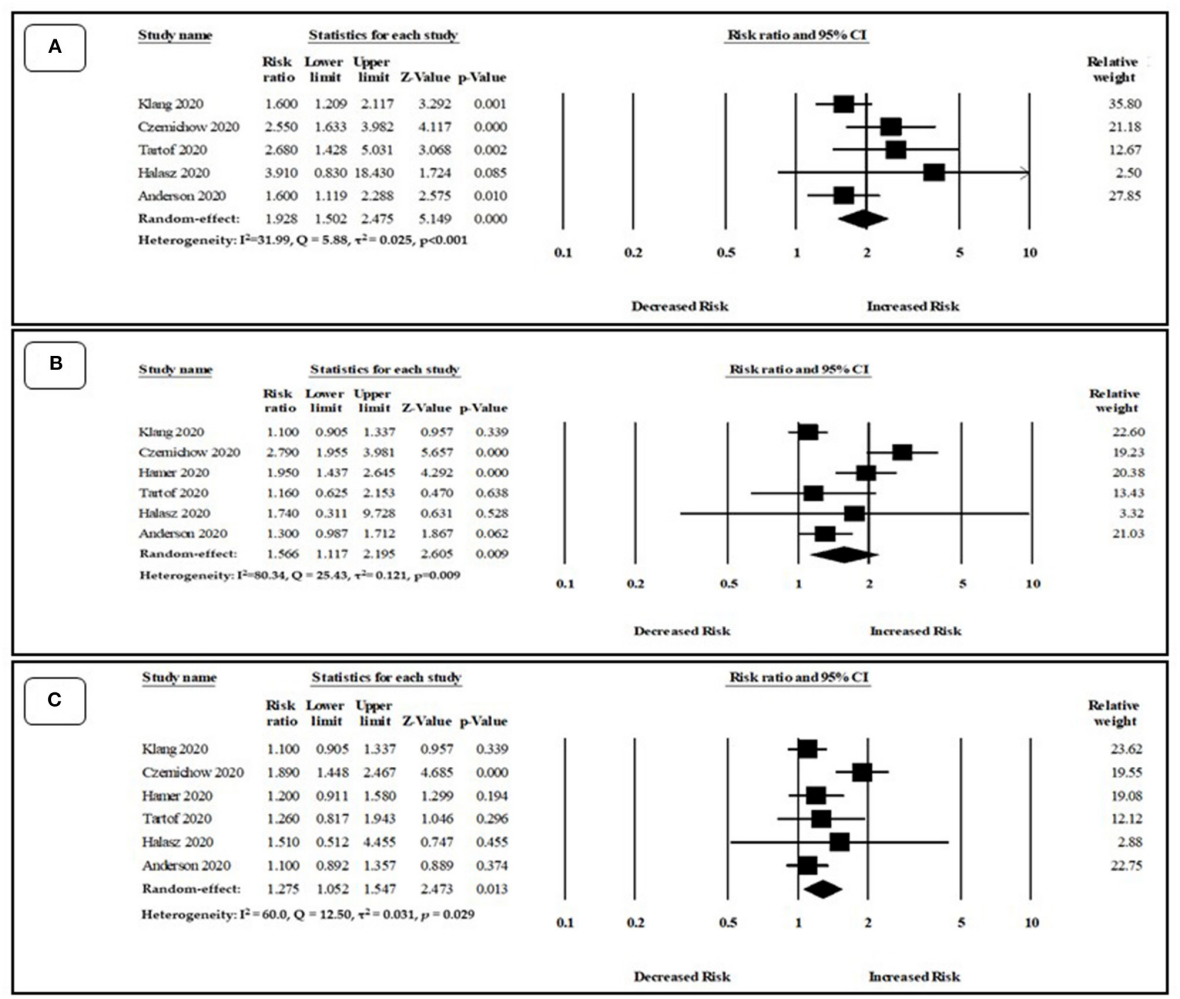
Association between **(A)** Class III, **(B)** class II, **(C)** class I obesity and risk of mortality among COVID-19 patients.

We also pooled RR to evaluate the risk of mortality based on three types of obesity such as class I, class II, and class III obesity. Class III obesity was strongly associated with an increased risk of mortality, with a pooled RR_adjust_ of 1.92 (95%CI: 1.50–2.47, *p* < 0.001). There was insignificant low heterogeneity among the studies (*I*^2^ = 31.99, *Q* = 5.88, tau^2^ = 0.02, *p* = 0.20) (Figure 4A). Class I and class II obesity also showed a strong association with an increased risk of mortality, with a pooled RR_adjust_ of 1.27 (95%CI: 1.05–1.54, *p* = 0.01), and 1.56 (95%CI: 1.11–2.19, *p* < 0.01), respectively (Figures 4B,C). There was significant moderate heterogeneity among the studies for Class I (*I*^2^ = 60.00, *Q* = 5, tau^2^ = 0.03, *p* = 0.02) and higher heterogeneity among the studies were observed for class II (*I*^2^ = 80.34, *Q* = 25.43, tau^2^ = 0.12, *p* < 0.001).

### Subgroup Analysis

Subgroup analyses are presented in Table 2. The 7 studies reported the risk of mortality among patients with obesity who were more than 65 years old. The pooled RR_adjust_ was 2.54 (95%CI: 1.62–3.97, *p* < 0.001) and heterogeneity among the studies was (*I*^2^ = 89.33, *Q* = 56.24, tau^2^ = 0.24, *p* < 0.001). Furthermore, six studies evaluated the risk of COVID-19 mortality among male patients and showed a significantly higher risk (RR_adjust_: 1.38, 95%CI: 1.25–1.51, *p* < 0.001). There was a lower risk of heterogeneity among the studies (*I*^2^ = 9.57, *Q* = 5.53, tau^2^ = 0.001, *p* = 0.35).

**Table 2:** Subgroup analyses.

	**Pooled estimate**			**Test of heterogeneity**		
	***N***	**RR with 95% CI**	***p*-Value**	**τ^**2**^**	***I*^**2**^**	***p*-Value**
**Demographic**						
Male	6	1.38 (1.25–1.51)	**<0.001**	0.001	9.57	0.35
≥65 years	7	2.54(1.62–3.97)	**<0.001**	0.24	89.33	<0.001
**Comorbidity**						
Diabetes	11	1.19 (1.07–1.32)	**0.001**	0.012	54.15	0.016
Hypertension	9	1.07 (0.92–1.25)	0.351	0.029	60.58	0.009
CKD	7	1.57 (1.29–1.91)	**<0.001**	0.036	69.18	0.003
CPOD	5	1.34 (1.18–1.52)	**<0.001**	0.004	16.12	0.312
Stroke	2	1.80 (0.89–3.64)	0.100	0	0	0.37
**Others**						
Smoking	6	1.13 (0.91–1.40)	0.242	0.027	46.57	0.09

*N, number of study; RR, risk ratio; CI, confidence Interval; CKD, chronic kidney disease; CPOD, chronic obstructive pulmonary disease. Bold value indicates subgroup and significant p-value*.

We also evaluated the risk of mortality among obese COVID-19 patients with various comorbidity such as diabetes, hypertension, CKD, COPD, and stroke. The pooled RR for COVID-19 mortality among patients with diabetes was 1.19 (95%CI: 1.07–1.32, *p* = 0.001). However, the pooled RR among the patients with stroke, CKD, COPD, and hypertension were 1.80 (95%CI: 0.89–3.64, *p* = 0.10), 1.57 (95%CI: 1.57–1.91, *p* < 0.001), 1.34 (95%CI: 1.18–1.52, *p* < 0.001), and 1.07 (95%CI: 0.92–1.25, *p* = 0.35), respectively.

### Risk of Bias

**Figure 5:**
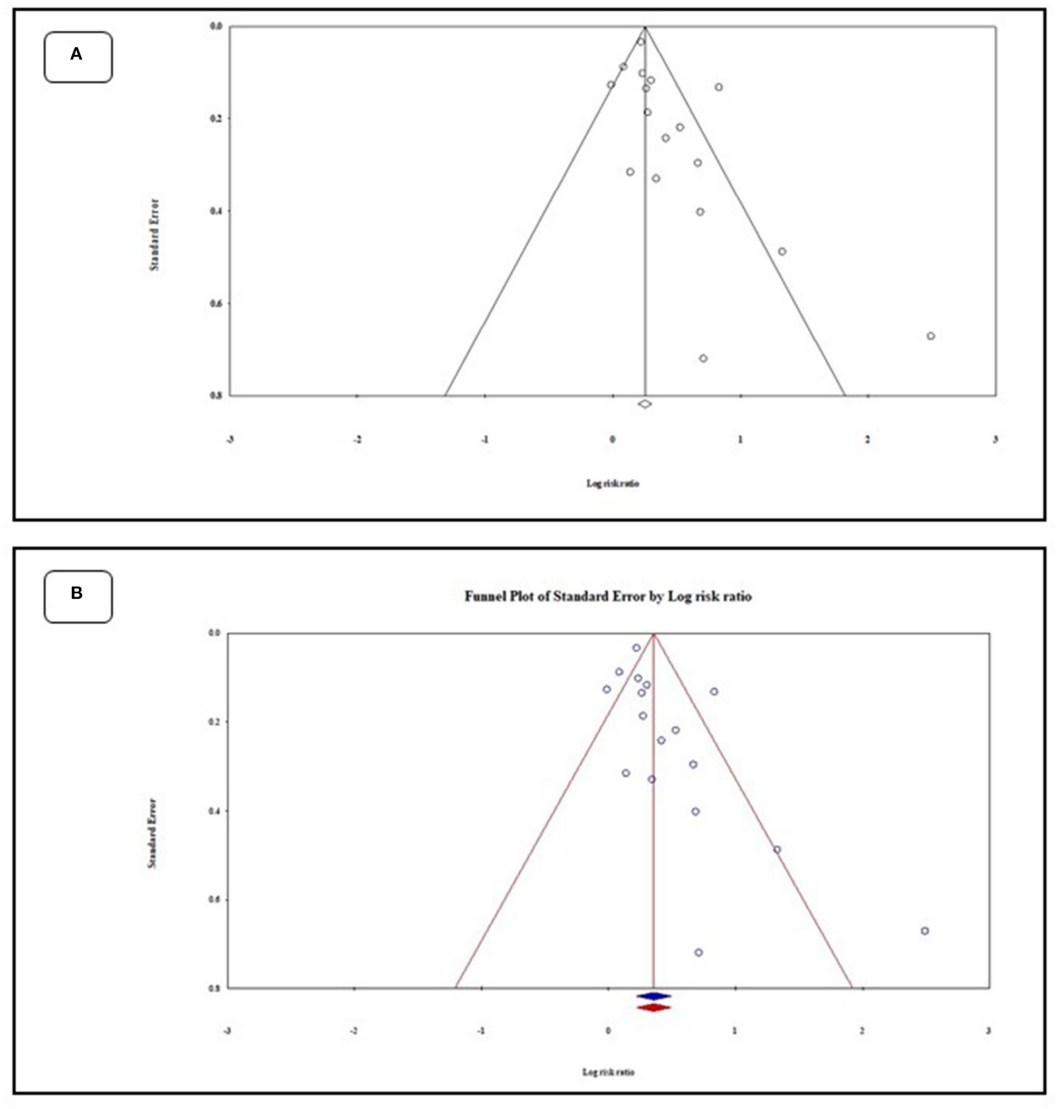
Funnel plots of meta-analysis before **(A)** and after **(B)** applying the fill and trim method.

Figure 5A depicts the funnel plot that indicates no publication bias among the studies. Egger's regression test was used to evaluate the funnel asymmetry, which showed no publication bias (*p* < 0.05). Moreover, Figure 5B shows the funnel plot with missing studies imputed by the trim and fill method. There was no missing study to be filled in the plot, and the overall log risk ratio became 1.42 95%CI (1.24–1.63).

## Discussion

###  Main Findings

This rigorous meta-analysis of 17 studies showed that obesity is associated with an increased risk of mortality among patients with COVID-19, especially patients aged more than 65 years. Furthermore, class III obesity patients observed a greater risk of mortality compared to class I and class II obesity. The risk of mortality was varied in COVID-19 patients with various comorbidities. Our findings may help to categorized patients at different risk groups and to make potential early prevention and treatment strategies possible.

### Comparison With Previous Studies

Our study findings are similar to those of two previous studies (41, 42). Hussain et al. (41) included 14 studies, but only 6 studies were used to assess the relationship between obesity and mortality in COVID-19. This study lacks statistical power in subgroup analyses and included no consideration of different types of obesity and risk of mortality of patients with COVID-19. Another study showed a positive relation, though lack of evidence because they included preprints (non-peer-reviewed articles), and there were no subgroup analyses (42).

### Biological Plausibility

Several biological mechanisms have been reported to explain the association between obesity and increase the risk of mortality. First, it is reported that both ectopic fat and COVID-19 are responsible for upregulation of pro-inflammatory, angiotensin II (ATII), and prothrombotics. Patients with obesity observe a decreased level of inflammatory adipokines, adiponectin, which is liked to an increased rate of ATII (43, 44). Similarly, coronavirus reduces the activity of ACE2 inhibitor, which leads to increase ATII level (45, 46). Higher levels of ATII might contribute to the progression of lung injury among patients diagnosed with COVID-19 by triggering NADH/NADPH oxidase system (47) and promoting contraction and vasoconstriction (48). Furthermore, an increased expression of inflammatory molecules enhances the production of cytokines [e.g., tumor necrosis factor alpha (TNF-α) and IL-6] (49), which are associate with alveolar damage and an increased rate of mortality (50).

### Public Health Implication

COVID-19 pandemic has created serious concern globally, and people are eagerly waiting for potential vaccines. There is no exact and effective treatment for this virus so far, and global morbidity and mortality thus increase day by day (51). COVID-19 shows a wide spectrum of symptoms; some patients recovered without complications. However, some patients affected by serious illness have needed to transfer to the ICU, require a prolonged hospital stay, and may even die (52). Elderly patients were more vulnerable in this disease because they have multiple diseases. A significant number of studies reported that elderly patients and patients with diabetes, stroke, CKD, and COPD are associated with bad outcomes (53, 54). Obesity, especially class 3 obesity, was associated with an increased rate of mortality among patients diagnosed with COVID-19. It is, however, not surprising because patients with obesity who had the H1N1 influenza virus also observed prolong hospitalization, mechanical ventilation, and death when it was calculated as an independent risk factor (55, 56).

Several population-based cohort studies reported that obesity is linked to increased comorbidity like diabetes, hypertension, and heart disease. However, the mortality rate among patients with obesity proportionally increased with BMI (57, 58). Moreover, obesity makes patients' conditions worse if patients develop infections by downregulating the inflammatory cascade. Hyperactivation of inflammatory pathways surge the level of cytokines, adiponectin, and leptin and distort both macro- and micro-vascular responses (59). Obesity is also associated with lung function impairment, which involves altering mechanics and airway resistance and decreasing gas exchange (60, 61). The findings of our study suggest that physicians should focus more on COVID-19 patients with obesity because this group of patients is at high risk of worse consequences. Moreover, our results highlight the need for vigilance, priority on testing, and an earlier start to treatment in obese patients with COVID-19. Previous studies also mentioned that obesity increased the rate of hospitalization, worsened patient conditions, and increased invasive mechanical ventilation in those diagnosed with COVID-19 (15, 62).

### Strengths and Limitations

Our study has several strengths. First, this is the first comprehensive and rigorous meta-analysis that assessed the association between obesity and the risk of mortality among patients with COVID-19. This meta-analysis included 17 studies, and only peer-reviewed articles were included for calculating the magnitude of risk because preprint articles have lots of room for improvement. Second, the risk of bias is low, and heterogeneity among the study is moderate. However, several factors showed higher heterogeneity among the studies. Third, this meta-analysis also evaluated associated factors that can contribute to a high risk of mortality. Finally, only adjusted effect size was considered to calculate pooled RR, which indicates strong evidence with a low risk of bias. Our study has several limitations that need to be addressed. First, we could not compare risk among patients aged <65 due to data constraints. Second, pneumonia is a high-risk factor for mortality among COVID-19 patients because only one study reported about this issue. This study showed the association between COPD and the risk of mortality among patients with COVID-19. Third, our study did not show the difference in the rate of mortality among various races and locations; this could have added value to the present evidence.

## Conclusion

We conducted a comprehensive systematic review and rigorous meta-analysis of studies reporting the risk of mortality among COVID-19 patients with obesity. Our study findings showed that obesity is associated with an increased risk of mortality among patients with COVID-19. Importantly, the risk of mortality was higher among class III obesity than class I and II obesity. Physicians should be aware of these risk factors and make a quick decision for intervention. Future studies are urgently needed to clarify the pathophysiological relationship between obesity and the risk of mortality among COVID-19 patients.

## Data Availability Statement

The original contributions presented in the study are included in the article/Supplementary Material, further inquiries can be directed to the corresponding author/s.

## Author Contributions

TP and MI: conceptualization. TP and HY: methodology. MI: Software, resources, data curation and writing —original draft preparation. ML, W-SJ, and Y-CL: validation. TP: formal analysis and visualization. M-HH: investigation. Y-CL: writing —review and editing and supervision. All authors contributed to the article and approved the submitted version.

## Conflict of Interest

The authors declare that the research was conducted in the absence of any commercial or financial relationships that could be construed as a potential conflict of interest.
